# HIV Resistance Prediction to Reverse Transcriptase Inhibitors: Focus on Open Data

**DOI:** 10.3390/molecules23040956

**Published:** 2018-04-19

**Authors:** Olga Tarasova, Vladimir Poroikov

**Affiliations:** Institute of Biomedical Chemistry, 10 building 8, Pogodinskaya st., Moscow 119121, Russia

**Keywords:** HIV, reverse transcriptase, resistance, computational prediction, amino acid sequences, nucleotide sequences, open data

## Abstract

Research and development of new antiretroviral agents are in great demand due to issues with safety and efficacy of the antiretroviral drugs. HIV reverse transcriptase (RT) is an important target for HIV treatment. RT inhibitors targeting early stages of the virus-host interaction are of great interest for researchers. There are a lot of clinical and biochemical data on relationships between the occurring of the single point mutations and their combinations in the pol gene of HIV and resistance of the particular variants of HIV to nucleoside and non-nucleoside reverse transcriptase inhibitors. The experimental data stored in the databases of HIV sequences can be used for development of methods that are able to predict HIV resistance based on amino acid or nucleotide sequences. The data on HIV sequences resistance can be further used for (1) development of new antiretroviral agents with high potential for HIV inhibition and elimination and (2) optimization of antiretroviral therapy. In our communication, we focus on the data on the RT sequences and HIV resistance, which are available on the Internet. The experimental methods, which are applied to produce the data on *HIV-1* resistance, the known data on their concordance, are also discussed.

## 1. Introduction

Acquired HIV drug resistance is an essential problem of highly active antiretroviral therapy (HAART). HIV reverse transcriptase (RT) is part of HAART and one of the most attractive targets for the HIV inhibition [1,2]. The antiretroviral drugs that inhibit HIV RT only allow decreasing the *HIV-1* replication but do not provide the full elimination of the virus [3]. Therefore, the development of new antiretroviral drugs is still of high interest due to the issues of safety and efficacy of the drugs, which are currently used in clinical practice [4].

An acquired HIV RT resistance occurs due to the high rate of mutations in a particular region of the pol gene, encoding the HIV RT amino acid sequences [5]. There are a lot of data on the relationships between mutations and their combinations in the pol gene and the combination of medicines prescribed to a patient. There is also information about activity of the nucleoside reverse transcriptase inhibitors (NRTI) and non-nucleoside reverse transcriptase inhibitors (NNRTI) approved by U.S. Food and Drug Administration for the HIV with particular variants of the RT. Most data on the relationship between amino acid substitutions in reverse transcriptase and acquired HIV type 1 resistance are available for the *HIV-1B* subtype. However, it was shown that response to therapy depends on a particular mutation as well as on the *HIV-1* type and subtype (for instance, *HIV-1B*, *HIV-1A*, *HIV-1C*, *HIV-1D*) [6]. In particular, it was shown that *HIV-1C* patients from Botswana who received HAART zidovudine and didanosine developed resistance predominantly through the 67N/70R/215Y pathway [7]. These mutations are different from zidovudine/didanosine-associated resistance mutations in *HIV-1B* (combination of mutations (M41L, L210W and T215Y) or (D67N, K70R and K219E/Q)). Other factors that influence the outcome of therapy include transmitted drug resistance, patients’ age, genotype, adherence to therapy, etc. [6,8,9]. The inhibition of *HIV-1* replication by the NRTI and/or NNRTI depends on the resistance to the viral subtype predominant in a particular patient. Therefore, it is clear that there is no direct relationship between specific mutations and their number and the outcome of therapy in an individual patient. Moreover, acquired *HIV-1* drug resistance might be a result of mutations and/or their combinations occurring de novo [10] and data on which are absent in the databases of the *HIV-1* resistant strains as well as in scientific publications. 

The analysis of data availability and results heterogeneity of the existing approaches to the study and prediction of the *HIV-1* drug resistance is essential for the development of new approaches in this field of knowledge. Systematic analysis of the initial biochemical and clinical data on the *HIV-1* drug resistance is of particular importance for development of a roadmap, which will lead to the (1) prediction of *HIV-1* resistance with high performance and (2) creation of new antiretroviral agents with high potential for *HIV-1* inhibition and elimination. Moreover, the development of methods that are able to predict *HIV-1* resistance based on amino acid or nucleotide sequences can be applied to the selection and optimization of antiretroviral therapy.

There are several reviews dedicated mainly to the computer-aided prediction of the *HIV-1* resistance caused by structure changes in *HIV-1* proteins [11,12], including web-services based on these methods [12,13]. For example, Martinez-Picado, J. and Martínez, M.A. described the investigation of experimental methods of the *HIV-1* resistance [14]. The perceptions of antiretroviral drug development in three spaces (biological, chemical and clinical) were considered in the study of Pauwells, R. [15]. In contrast to these reviews, we consider three mandatory parts of *HIV-1* RT-associated resistance prediction: (1) experimental methods; (2) the data on the *HIV-1* resistance freely available on the Internet, and (3) the methods of the *HIV-1* resistance prediction with the focus on the data. We assume that there is a close relationship between the experimental data concordance and the possible differences in the results of prediction. In order to consider the level of concordance at the stage of experiment and though computational predictions we have analyzed each source of the data using the information about it available in any database of scientific information. We explored the interface of the data sources for RT sequences and summaries and analyzed the data that can be downloaded from them. Availability of data on the *HIV-1* resistance for computational experiments including the possibility of using the application programming interface (API) to collect sequences and the data associated with them are discussed. Taking into account the current state of the data sources, experimental and computational studies on the resistance due to structural changes in the *HIV-1* RT, we present the outlook on the *HIV-1* drug resistance analysis and prediction to design novel pharmaceutical agents with high antiretroviral potential. 

## 2. Results and Discussion

### 2.1. Experimental Methods of HIV-1 Resistance Detection and Concordance between Their Results

There are two types of experimental methods for estimating the susceptibility of *HIV-1* samples replicated in patient’s plasma: (1) genotypic and (2) phenotypic methods. Both approaches can determine the nucleotide sequences of *HIV-1* isolate. However, phenotypic assays can also detect the susceptibility of *HIV-1* to an antiretroviral drug while genotypic assays are used to detect mutations for which association with the *HIV-1* resistance has been already determined. Therefore, phenotypic assays are important for the analysis of genotype-phenotype relationships, which are used to further interpret the results of genotype assays.

Genotypic assays are based on sequencing of nucleotide sequences of the *HIV-1* genes with identification of patterns of HIV mutations associated with antiretroviral resistance. The method is more commonly used in clinics since it is more affordable and requires less time in comparison to phenotypic assays. However, there are several clinical studies where the utility of genotypic assays for therapy management has been demonstrated [16,17]. Nevertheless, genotypic assays cannot be used for modeling of the *HIV-1* resistance based on nucleic acids since they do not provide any information about the relationship between the nucleic/amino acid sequences and the inhibitory activity of drugs against a specific variant of *HIV-1*.

Phenotypic assays include methods that can be performed using either purified RT enzyme or cell lines. Most of these methods assess kinetics of the *HIV-1* replication in the cell lines inoculated with *HIV-1*. Typically, the replication kinetics can be assessed for a single *HIV-1* variant or for two viral variants mixed to produce an infection. Resistance to a specific drug can be estimated in the presence of this antiretroviral drug [18,19]. Determination of replication kinetics for the growing concentrations of an antiretroviral drug provides an opportunity to determine the dose-effect relationships with subsequent calculation of the IC_50_ value. The ratio of IC_50_ of a mutant *HIV-1* variant to that of a wild-type *HIV-1* is calculated to obtain the Fold Ratio (FR) value. 

Currently, there are several commercially available test systems, the most known of them are Antivirogram™ (Virco™ [18]) and Phenosense™ (Virologic™ [19]). Both assays are based on the recombinant virus assay technologies. Phenosense™ is based on usage of vector constructed using the NL4-3 infectious molecular clone of *HIV-1*. *HIV-1* envelope gene is replaced by vector containing a luciferase expression cassette. Protease (PR, entire sequence) and reverse transcriptase (part of a sequence) are amplified from plasma *HIV-1* RNA and inserted into the vector using specific restriction sites. Recombinant viruses are generated by the transfection of the HEK 293 cell line [20] using vector DNA. To measure PR and RT activity, the luciferase activity detected in the cells infected is measured.

Antivirogram™ uses pGEMT3ΔPRT plasmid. *HIV-1* RNA from the patient’s plasma contains the *HIV-1* PR and RT-coding sequence. The pool of PR-RT-coding sequences is then cotransfected into CD4+ T lymphocytes (MT4) with the pGEMT3ΔPRT plasmid leading to the generation of chimeric viruses containing PR and RT coding sequences (derived from *HIV-1* RNA in plasma). The susceptibilities of chimeric viruses to all currently available RT and/or PR inhibitors are determined by an MT4 cell–3-(4,5-dimethylthiazol-2-yl)-2,5-diphenyltetrazolium bromide-based cell viability assay (MT4-MTT)-based CPE protection assay [18]. 

Many studies have been dedicated to the reproducibility of Antivirogram™ and Phenosense assays and correlation between them [21,22,23,24,25,26], in general, reporting higher sensitivity of the PhenoSense assay in comparison to those for Antivirogram™ at detecting resistance to drugs with relatively small FR values. Higher reproducibility was also shown for Phenosense™ versus Antivirogram™ assay [25,26,27,28]. Demeter, L. and Haubrich, R. reviewed the earliest publications including [29] in their study [30]. The authors of the original papers reached different conclusions about the concordance between the two test systems. In particular, in Qari, S.H. et al. reported that Antivirogram™ and Phenosense™ correlated well (Pearson correlation coefficient was 91%) [21]. They used 50 plasma specimens, from which “data for 12 to 15 drugs were available by both assays for 38 specimens” yielding 529 pairs of results. The authors used the plasma specimens selected at the US Centers for disease control and prevention. Ross, L. et al. used 70 specimens tested against a panel of 14 drugs and determined the concordance between the two test systems [25]. The authors have reported a high overall concordance (89%). The kappa statistic (a measure of agreement that corrects for the chance-expected agreement and observed one) was 0.62 and corresponded to a good agreement. As the authors of the previous study, Ross, L. et al. used the specimens obtained from a cohort of patients in the hospital [25]. Wang, K. et al. evaluated the correlation coefficients of the isolates retrieved from the Stanford University HIV drug resistance database using Spearman rank correlation coefficients (rs) [26]. The results reported for 14 drugs are as follows: rs values ranged from 0.018 for saquinavir (SQV) to 0.551 for lamivudine (3TC), with a median of 0.290 [26]. Therefore, these results were contradictory to those of Qari, S.H., et al. [21]. Wang, K. et al. explained this fact by the following reasons: for a majority of cases, Qari, S.H., et al. used the data set consisted of the drug susceptible samples that can partially shift the data set to the samples having high concordance between the values belonging to a class susceptible/resistant. In the publication of Wang, K. et al. [23], the authors used the complete data set from HDRDB and calculated the concordance and correlation coefficient for PR and RT inhibitors. They reported rs = 0.37 between two assays for RT inhibitors, noting high reproducibility of both assays. They also analyzed the relationship between the concordance and the cutoff value for making a decision on the drug resistance. One of the general conclusions made by Wang, K., et al. is that there was a great dependence of the concordance value on the cutoff value and on the fraction of susceptible samples in both data sets.

There are several common drawbacks for both genotype and phenotype assays limiting the usage of their results in clinics as well as computational algorithms of the HIV resistance prediction. First, both type of assays can produce mixtures from the protease and RT isolates retrieved from patients receiving HAART. Parkin, N. et al. have shown that in contrast to genotypic assays, phenotypic assays do not detect resistance if a mutation is present in a minority of the *HIV-1* variants [31]. Second, typically none of these two assays can produce an entire sequence of the reverse transcriptase after sequencing. Other limitations of phenotypic assays are associated with a complex nature of *HIV-1* resistance, and the measure of *HIV-1* resistance determined in in vitro/ex vivo experiments cannot be the same as in vivo because the virus is the inoculant especially inserted in cell cultures for replication. At the same time, experiments carried out in cell lines differ from those in vivo conditions because of different protein profiles and tissue differentiation.

Despite the existing limitations, currently, phenotypic *HIV-1* susceptibility assays are the most important source of the *HIV-1* sequences and the relationships between specific *HIV-1* sequence and viral phenotype. 

The data obtained in genotypic and phenotypic *HIV-1* susceptibility assays are stored in the *HIV-1* sequences repositories that will be considered further.

### 2.2. HIV Sequences Repositories

#### 2.2.1. NCBI Nucleotide (GenBank) and NCBI Protein

**Figure 1:**
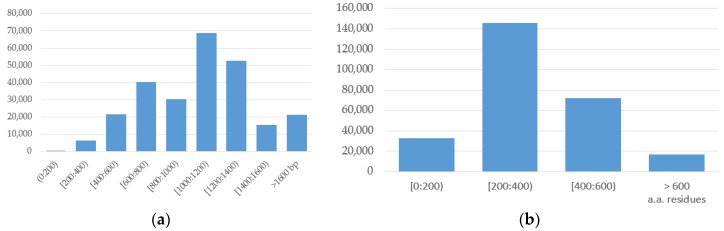
Distribution of the (**a**) *HIV-1* nucleotide sequences depending on the sequence length (NCBI Nucleotide (GenBank) database); (**b**) *HIV-1* amino acid sequences depending on the sequence length (NCBI Protein database).

NCBI Nucleotide [32,33] is the largest repository of nucleotide sequences [32] integrating three major sources of genetic sequences: the DNA data bank of Japan, the European Molecular Biology Laboratory and the National Center for Biotechnology Information, USA. As of 15 March 2018, it contains more than 257,000 sequences of *HIV-1* of various lengths (Figure 1a). There are several links to other repositories, including the NCBI protein database, which contains the translation of nucleic acid into more than 267,000 amino acid sequences of various lengths (Figure 1b).

The data can be easily retrieved from various NCBI databases, including the NCBI Nucleotide and NCBI protein using REST protocol [34] as well as especially developed web-services which are based on REST technology [35] presented the integrated web-based APIs for interoperable bioinformatics web-services. In particular, using this approach one can easily retrieve a set of GenBank Accession Numbers for *HIV-1* reverse transcriptase. GenBank Accession Number can be further used to access a related identifier from NCBI Protein database and translation of a protein related to it. It is impossible to determine the association between (i) nucleotide sequences from either NCBI nucleotide database or amino acid sequences from NCBI protein and (ii) level of resistance for the HIV-variant containing a particular sequence, because of the absence of these data in both NCBI nucleotide and NCBI protein databases. We would like to emphasize that inclusion of cross links to the databases of *HIV-1* resistance could be very useful for investigators in the field of *HIV-1* resistance.

#### 2.2.2. Los Alamos HIV Sequence Database

Studies of *HIV-1* resistance allowed revealing that the most important reasons for the resistance are mutations in the genes coding *HIV-1 PR*, *HIV-1 RT*, and gp41 envelope protein. These studies gave a start to identification of drug resistant mutations in clinical isolates.

Accumulated data about amino acid sequences provide the basis for the development of databases containing the nucleic and amino acid sequences of the main *HIV-1* target proteins such as integrase, protease, and reverse transcriptase. In most cases, the source of these data is the genotyping of clinical isolate with subsequent establishment of three types of data. First, there are the relationships between treatment with certain *HIV-1* antiretroviral drugs and mutations in *HIV-1* proteins (genotype-treatment). Second, there are the relationships between the determined nucleic/amino acid sequences of any *HIV-1* gene/protein and in vitro experiments on the drug susceptibility (genotype-phenotype). Third, there are the relationships between the nucleic/amino acid sequence of any *HIV-1* gene/protein and viral load after the treatment regimen (genotype-outcome). All the data types are important for the HIV-positive patient management since it was shown that the availability of the drug resistance data to the physicians influences the therapeutic outcome [13]. Typically, the computer-aided systems for genotypic-resistance interpretation are based on one or more types of data. There are two freely available repositories of the *HIV-1* sequences: the Los Alamos HIV sequence database and the Stanford University HIV drug resistance database. Also, these two databases contain nucleic or amino acid sequences of hepatitis B and C, data on the treatment schema of patients, whose biological material was used to obtain clinical isolate, data on the biological peculiarities of viruses, their evolution and immunological properties (for instance, the epitopes sequences).

The Los Alamos HIV sequence database (part of Los Alamos HIV databases), which was developed and maintained in the Los Alamos National Laboratory (LANL), Los Alamos, NM, USA, initially was created as the repository of the *HIV-1* and HIV-2 sequences and Simian Immunodefficiency Virus (SIV) sequences, exported from GenBank. 

Amino acid sequences were downloaded from GenBank database automatically using the text-parsing scripts. After downloading, the sequences were annotated by experts based on the information from the full-text publications and communication with the authors of these publications, which is given as a reference in GenBank. The data associated with amino acid sequences (such as the properties of the virus, sequencing method, co-receptor usage etc.) should be also annotated. The experts annotated only the sequences with length more than 280 bp, or those spanning a complete gene, or representing an unusual subtype or a rarely sampled geographical region [36].

The Los Alamos HIV sequence database covered the amino acid sequences alignments and nucleic acid sequences alignments curated by experts. Only one sequence from a patient was included in alignment to avoid biasing the data with large sequence sets from a few sources [36]. In this case, part of the data will be lost because one patient can be a source of innumerable variants of *HIV-1* distinct from the predominant variant. The alignments are based on the codons matching because frameshifts, insertions, and deletions are very common in *HIV-1*.

In addition to the expert-curated pre-calculated alignments, a researcher can download full-length sequences, which can be further aligned by users. Many web-services of the Los Alamos HIV sequence database allow solving multiple tasks. Kuiken, C. describes them in details [36]. We should emphasize that they are dedicated mainly to the analysis of epitopes, alignment, extraction of the GenBank identifiers etc. However, there are no services that provide either genotype-phenotype interpretation or genotype-resistance interpretation.

Earlier, the Los Alamos HIV Resistance Mutation Database was developed as a part of the Los Alamos HIV sequence database. As of 25 May 2015, this database contained about 300 records with amino acid replacements in different positions and the comments about the resistance of a particular sequence variant to a certain antiretroviral drug. The most frequent positions associated with drug resistance were positions 101, 103, 106, 108, 179, 181, 184, 190. However, today the Los Alamos HIV Resistance Mutation Database is no longer maintained. The data on mutations associated with the *HIV-1* resistance may be found in reviews published in the framework of the database guidelines every year; for instance, see a review of Clark, S.A. et al. [37].

Currently the Los Alamos HIV sequence database does not include special web resources for the *HIV-1* resistance mutations and the data on a particular variant resistance. Thus, information from the database cannot be used in any approaches related to the HIV resistance prediction. Nevertheless, this database can be used for studies of *HIV-1* evolution as well as for the search for a particular *HIV-1* variant to estimate its prevalence in certain geographical region. In addition, there are a lot of tools allowing working with HIV sequences, in particular, tools for sequence alignment; tools to construct phylogenetic trees; tools to create specific epitopes etc.

#### 2.2.3. The HIV Oligonucleotide Database (HIVoligoDB)

The database contains oligonucleotides of HIV genes reflecting the most important for treatment and diagnosing of HIV/AIDS [38]. The selection of the most conserved HIV regions is also available. Oligonucleotides were partially collected from NCBI as well as retrieved from literature. As of 8 April 2018, the database consists 380 *HIV-1* nucleotides collected from 54 scientific publications. The HIVoligoDB might be used in biochemical and experimental studies of *HIV-1* mainly, however, it also can be used to determine are of interest of *HIV-1* variants’ sequences, as well as evolutional studies and determining the most conserved regions of *HIV-1* sequences.

#### 2.2.4. Stanford University HIV Drug Resistance Database

The Stanford University HIV drug resistance database (HDRDB) contains the nucleic and amino acid sequences of the reverse transcriptase and protease, the data on all the three types of relationships, described above, i.e., genotype-treatment, genotype-phenotype and genotype-outcome correlations that are available by different queries [39]. The data summaries including the most frequent mutations leading to viral resistance to NRTI and/or NNRTI are available in different formats. According to the data summaries of HDRDB (April, 8, 2018) the major drug resistance positions associated with the resistance to NRTIs are: 41, 65, 70, 74, 75, 115, 151, 184, 210, 215, the major positions associated with the resistance to NNRTI are: 100, 101, 103, 106, 181, 188, 190, 230. The data are in agreement with the interpretation of resistance in the experimental procedures available for several mutations such as K103N, Y181C, Y215T, M184V, M41L [40,41,42,43,44,45,46,47,48,49,50]. Although for many other mutations X-ray crystallographic data or NMR data on a three-dimensional structure are not available, the data based on biochemical experimental studies are present. Mutations in the position 115 occur rarely in comparison with other positions, according to the Stanford HIV RT and PR sequence database. 

The data on genotype-treatment correlations are available through the query, which may be executed using the form given at the website. The users can specify either the number of drugs included in the treatment regimen and their names or the codon, where the mutation occurred, and get the results. There is a possibility of creating advanced queries, where the selection of drugs after switching to the other treatment regimen is available. The data summaries on genotype-treatment data are summarized in graphical format. In addition, the users can download the reference dataset containing the data on Isolate Name (unique identifier of the clinical isolate), GenBank Accession Number, Region, Year, Subtype of the isolate and a list of drugs included in the treatment regimen, the data on the amino acid and nucleic sequences of RT, PR and integrase. As of 8 April 2018, (the last update is on 18 May 2011), the reference data set on genotype-treatment correlations contained the records of 43,995 isolates with RT sequences available for downloading. GenBank Accession Numbers can be used for retrieving of annotated nucleic acid sequences and associated proteins from NCBI Nucleotide and Protein databases but Accession Numbers are available for 35,798 isolates (about 81% of the complete data set). We should emphasize that by default the data on genotype-treatment correlations do not cover the complete amino acid sequences, but the nucleic acid sequences are available for all the isolates included in the reference data set. However, 23,244 isolates (about 50% of the whole set) contain symbols (“X”, “Y” etc.), distinct from those encoding nucleic acids “A”, “T”, “G”, “C” and “U”, which makes it difficult to accurately interpret a nucleotide sequence and/or translation it into the amino acid sequences. The data on genotype-treatment correlations may be used in the systems of automated genotype-resistance interpretation with a suggestion about the importance of mutations detected in a patient receiving a medicine for the drug resistance. One of the most common restrictions of this type of data is representing of only a subset of a pool of mutations, which potentially can be selected during the consumption of drug by patients [13]. Difficulties with the interpretation of nucleic acids sequences due to occurring extraneous symbols in the sequences and unavailability of the GenBank Accession Numbers for a part of the data set are probably sources of noise when the data are used in computational approaches. Nevertheless, an overlap between genotype-treatment and genotype-phenotype data from HDRDB may be useful for the creation of data sets and their usage in HIV resistance prediction.

The data on genotype-phenotype correlations are available in a form of the query, where a user can select any codon with potential mutation, name of seven NRTI or five NNRTI and one of the susceptibility test systems, Phenosense (Virologic™) and Antivirogram (Virco™), or all of them. The data summary is available as a table containing over 500 patterns of the major drug resistant mutations with the median fold resistance to each of four drugs classes (zidovudine, tenofovir, abacavir, lamivudine). Downloadable data sets are also available and include the results of in vitro susceptibility of isolates performed either using Phenosence (Virologic™) or Antivirogram (Virco™) method. These data sets contain the data on the sequence identifier (SeqID), patient identifier (PtID) and isolate identifier (Isolate Name), HIV subtype (Subtype), isolate type (i.e., clinical or laboratory isolate, type), fold rate (FR), reflecting the susceptibility of HIV variant in the isolate compared to the wild-type. The FR values are given for ten drugs including NRTIs and NNRTIs. The sequences are available in the reference data sets. However, similar to the genotype-treatment data set, the complete amino acid sequences are not present; typically, there are only first 440 amino acid residues. Nucleotide sequences are not available at all. There are consensus sequences of the aligned wild-type *HIV-1* with the indicated replacements in the several different positions in each sequence, this consensus sequence is different from the wild-type *HIV-1* sequence available at NCBI repositories (NP_057849.4) by several amino acid positions: 169, 261, 319, 324, 423, 448, 507, 515, 530, 559, 566. (For details, please, see Figure S1 of Supplementary Materials, the alignment was made using ClustalW package [51] at the web-site of the European Bioinformatics Institute (EBI)).

The positions with different amino acid residues are not commonly occurred in the regions with high mutation prevalence and hence can be considered as a consequence of a general viral variability.

Genotype-phenotype relationships should have a crucial importance for the purposes of computer-aided drug design since they provide the most detailed information about particular mutations and affinity of a certain drug to the isolate containing these mutations. For this reason, using the in-house developed Python script we estimated the number of sequences, where a mixture occurs in at least one position, assumed that the presence of any mixture could be a source of noise for computational experiments.

**Figure 2:**
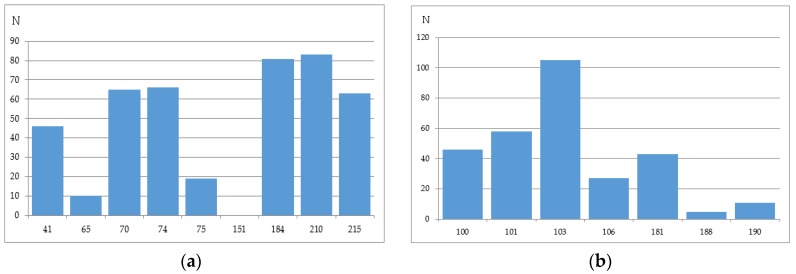
The number of mixtures occurring in the major drug resistance positions in the initial data sets obtained using the Phenosense assay: (**a**) for main NRTI-associated mutations; (**b**) for main NNRTI-associated mutations.

**Figure 3:**
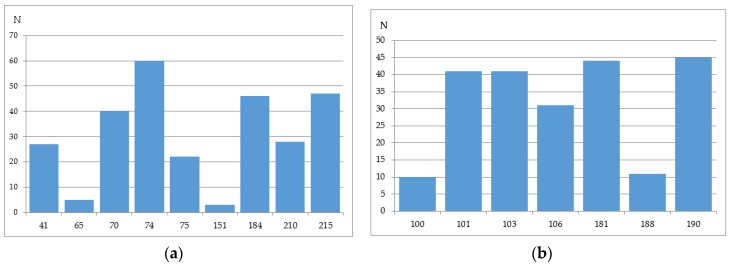
The number of mixtures occurring in the major drug resistance positions in the initial data sets obtained using the Antivirogram assay: (**a**) for main NRTI-associated mutations; (**b**) for main NNRTI-associated mutations.

We have analyzed the Phenosense (1984 records) and Antivirogram (1663 records) reference data sets and estimated the rate of sequences containing a mixture in at least one position, a number of mixtures occurring in the major drug resistance positions, and the total amount of records with a particular amino acid in each of the major drug resistance position. Some mixtures occurring in the major drug resistance positions in the initial data sets obtained using the Phenosense and Antivirogram assays are given in Figure 2a,b and Figure 3a,b. From Figure 2 and Figure 3 it is clear that the number of mixtures is not high for all major drug resistant positions; therefore the data on the residues at these positions can be used as descriptors for classification of amino acid sequences into susceptible and resistant variants.

**Figure 4:**
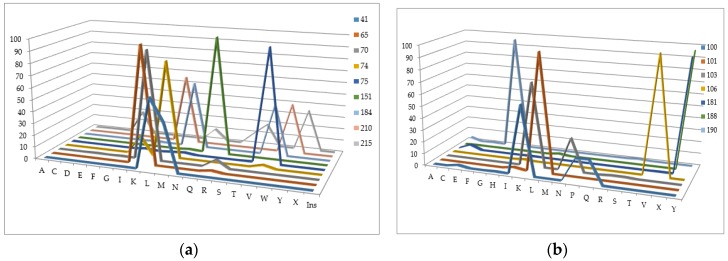
Distribution of amino acid residues occurring in the major drug resistance positions for the data set containing the drug susceptibility obtained using PhenoSense (Virologic™) assay (**a**) for NRTIs; (**b**) for NNRTIs.

**Figure 5:**
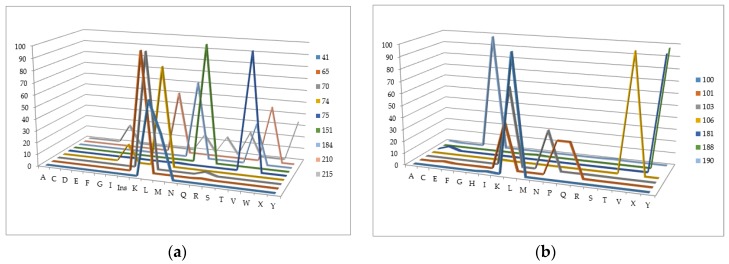
Distribution of amino acid residues occurring in the major drug resistance positions for the data set containing the drug susceptibility obtained using Antivirogram (Virco™).assay (**a**) for NRTIs; (**b**) for NNRTIs.

The number of sequences with at least one mixture indicated is 780 for the Phenosence data set (38% of the initial set size), and 1663 for the Antivirogram data set (65% of the initial set size). Further, we generated all sequence combinations for the Phenosense and Antivirogram data sets and calculated the number of sequences with a certain amino acid in each major drug resistance position (Figure 4a,b and Figure 5a,b).

According to the data in Figure 4 and Figure 5, we can make several conclusions. First despite an uncertainty being a result of the mixtures occurrence in the amino acid residues in the major drug resistant positions, the general patterns of particular amino acid residues distribution in those positions are similar for Phenosense and Antivirogram data sets. Second, amino acid residues in the major drug resistant positions are biased to the residues occurring in wild-type sequences for both Phenosence and Antivirogram sets. The sets, which may be used in the computational procedures, are highly imbalanced that may lead to a certain accuracy decrease when the HIV drug resistance should be predicted.

Finally, since the complete amino acid sequences are not provided for the genotype-phenotype correlations sets, we tried to match the sequences of isolates from the Phenosense and Antivirogram sets and those from (1) Genbank and (2) data sets on the HDRDB genotype-treatment correlations. We used the Isolate Name as a field to identify an overlap between the sequences from HDRDB and the GenBank. AccessionID (GenBank Accession Number) was further used to retrieve the complete sequences from GenBank. As a result, 399 unique sequences with known GenBank Accession ID were identified (they are provided in Supplementary Materials). We assume that these matched sets with the complete sequences from GenBank and the data on the susceptibility of *HIV-1* variant may be used in computational experiments instead of incomplete sequences to obtain the most accurate results of modeling. An overlap sets between genotype-phenotype and genotype-phenotype relationships included 1030 unique isolates as an overlap for Antivirogram-getotype-treatment and 698 unique isolates for Phenosense-getotype-treatment (they are also provided in Supplementary Materials). We propose that the overlapping data may be used as the reference sets to determine the relationships between treatment, mutations and the resistance. All the data sets are available in Supplementary materials in text format.

Genotype-clinical outcome correlations are available as the data on the HIV RNA level in patient’s plasma or CD4+ cells count in the patient’s blood. The data sets from clinical studies containing genotypes, treatment schemas, plasma *HIV-1* RNA levels, and CD4 counts are available for download for research purposes only. The summaries of clinical studies are presented.

The data on the sequences of different *HIV-1* variants are available in several publicly accessible databases. Typically, the number of sequences in the sequence databases is over a thousand. However, for some of *HIV-1* sequences, the data on susceptibility are not accessible. Relatively small data sets containing the data on *HIV-1* susceptibility are still important for clinicians and researchers since allow them to determine the prevalence of the *HIV-1* variant and get clinically relevant summaries of the *HIV-1* susceptibility. Despite the restrictions described above, the *HIV-1* sequences included in the freely accessible repositories is an important source for various computational methods for *HIV-1* resistance prediction.

### 2.3. Computational Methods of HIV-1 RT Associated Resistance and the Level of Concordance between Them

Currently, a variety of computational approaches directed to the prediction of *HIV-1* susceptibility to the anti-HIV drugs is available. Typically, either one type of the relationships (“genotype-treatment”, “genotype-phenotype”, “genotype-outcome”) or the combinations of them are used for the prediction [13]. Many of these approaches [52,53,54,55,56,57,58] are directed not only to the prediction of the susceptibility of amino acid/nucleotide sequence of *HIV-1* to a certain antiretroviral drug, but also include the prediction of probability success. They may be considered as the genotypic interpretation systems [11,12,13,59]. Zazzi, M. et al. in their review [12] have also proposed the term “treatment optimization tools” to distinguish between the systems directed onto the prediction of *HIV-1* susceptibility for a single drug and the systems that provide help to clinicians with the treatment management and can only provide the probability of a certain outcome. In our review we mainly focus on the methods, where the *HIV-1* resistance, associated with RT can be predicted and describe the accuracy of prediction and concordance between them based on the earlier studies [11,12,13]. 

The existing approaches use as input data either the nucleotide sequence [55,56,57,58] of *HIV-1* genes or amino acid sequences of *HIV-1* protein [60,61,62,63]. All the approaches to the *HIV-1* resistance prediction can be divided in two general groups depending on the principal algorithm: (1) rule-based and (2) statistics-based and machine learning computational methods. There are several sophisticated approaches, where several machine learning algorithms in combination with additional data (i.e., treatment history [58] or the chemical structure of drugs [61] may be used at these different steps. Depending on the data sets used for training or rules preparation the methods for *HIV-1* RT associated resistance are based either on proprietary data or the freely available data from HDRDB. For some approaches web-services have been developed, other methods have been described in the scientific publications. All existing approaches have completely different output data: (i) the estimated probability of the treatment success; (ii) predicted FR value; (iii) prediction of the specific susceptibility levels (from two to five for distinct approaches). In Table 1, the algorithms of the *HIV-1* resistance prediction are listed with the information about the source of the data sets, used for the training (rules preparation), principal algorithm and output.

Several studies have been dedicated to the investigation of various computational genotypic interpretation systems [11,12,64,65].

Three studies [11,12] used the sets of clinical isolates to compare the commercially available VirtualPhenotype system to the one or more rules-based systems. They reported high levels of concordance, except for those nucleoside reverse-transcriptase inhibitors with a narrow range of observable phenotypic susceptibility levels; these were more likely to be considered resistant by the rules-based systems.

In the review [11] focused on expert-based systems, a dozen academic systems were studied, from which six are freely available systems. The authors also analyzed the concordance between four systems (ANRS, HIVdb, Rega, Tru-gene) on the basis of the results obtained by Ravela, J. et al. earlier [70]: 4.4% of completely discordant results; 29.2% partially discordant and 66.4% of concordant results. Liu, T.F. and Shafer, R.W. noted several main reasons of comparatively low concordance [13]. First, it is the usage of different data types for the training (genotype-phenotype, genotype-treatment or genotype-outcome); second, there is a variety of output data, in particular, the number of susceptibility levels, and finally, there are differences between the algorithms (in general, the algorithms based on the mutation penalties are more rigorous than the rule-based systems that used Boolean expressions).

In the comprehensive review of Prosperi, M. and de Luca, A., fourteen distinct systems have been considered [11]. Authors [11] have described the systems classification and the criteria of their validation; they noted that due to a great body of data on the *HIV-1* isolates sequences, it is possible to compare different systems using the prediction for these data as a comparison. In their review the authors have estimated the concordance between the classification results of HIVdb, Antiretroscan, ANRS, and Rega [11]. The concordance was obtained for 18 drugs, including nine RT inhibitors and eight protease inhibitors. The sequences of pol gene retrieved from the Los Alamos HIV repository were used as input data. Prosperi, M. and de Luca, A. reported a high level (90%) of full agreement between the four methods for both B and non-B *HIV-1* subtypes. These results are particularly discordant (do not correspond) with those reported earlier [70]. We assume that the difference between the results may be explained by the difference in the data sets used in both studies and different systems used for the comparison (one system was different: Tru-gene in the study of Ravela, J. 2003 [70]; and Antiretroscan in the review of Prosperi, M. and de Luca, A. [11]. There are the differences between the results of the two studies, however, we should emphasize that both of them were focused on the rule-based algorithms, and we suppose that the concordance between the rule-based approaches and the machine-learning methods may be much lower due to the differences in the algorithms. 

In the review of Zazzi, M. 2016, the compared algorithms were divided into two groups: (1) experts’ rule-based (Rega, HIV-Grade, AntiRetroScan (AntiViroScan)) and (2) treatment optimization tools (geno2pheno, EuResist, HIV-TRePS) [12]. They estimated the concordance between the algorithms included in each group with respect to seven drugs, including two NNRTIs, three integrase inhibitors, and five protease inhibitors. The authors [12] reported comparatively high discordance for NNRTIs (3.4%) but overall discordance was very low. The authors described several issues considering the concordance of different treatment optimization systems because of different criteria of virological output, the mutations included in predictions and the time of treatment. They reported that the concordance level may be different depending on the point of outcome selection. 

In addition to the difficulties with the concordance estimation and interpretation between different methods reported earlier [12,13], it looks reasonable to analyze the impact of experiment on the discordant results of HIV resistance prediction algorithms as three key features (input data, core (algorithm) and output) have already been widely discussed.

First, different computational methods use different data sets as the input, which can influence the concordance at this level. Either nucleotide sequence encompassing the region of interest is used or amino acid sequence of the RT protein/part of protein can be used. Differences in the test systems (i.e., Antivirogram, Phenosense etc.) used for obtaining experimental data, are important. Also, one could expect the differences in the prediction results since different methods use their own data sets with various coverage of pol gene. In our point of view, it is difficult to use the translation of nucleotide sequence as the input data for the methods where amino acid sequences are used as the input due to untranslatable symbols and mutations leading to the frameshifts associated with *HIV-1* resistance [59,71]. Second, either proprietary data or freely available data can be used as the input. Third, we can assume that the discordant results of the prediction may be due to the use of different FR values at the prediction stage. The results obtained by different computational genotypic interpretation systems can be discordant because of two reasons: (i) the usage of different cutoffs for a particular method and (ii) the impossibility of applying specific cutoffs for the sequences selected for comparison purposes, if we do not know the cutoff that was used for training set preparation. Finally, one possible reason for differences in the results obtained by different algorithms is the complexity of *HIV-1* as an organism. The general inconsistency of the data on *HIV-1* activity testing might be a cause of the algorithms inconsistency as we considered and reported earlier [72].

We have revealed that multiple studies on prediction of *HIV-1* resistance to antiretroviral drugs are based either on open data or on proprietary data on *HIV-1* sequences and the resistance of particular *HIV-1* variants. Despite some uncertainty of open data, it is clear that researchers might use them in various scientific studies due to (1) the availability of the data on the Internet and (2) comparatively high amount of data, collected worldwide, which could be integrated and processed in new computational algorithms. We have observed the general applicability of open data to research studies and briefly discussed its use in the computational *HIV-1* resistance prediction algorithms. To enlarge the view of possibility to use open data in computational biology and cheminformatics, aspects of the possible open data applications to *HIV-1* resistance prediction in clinical practice and new drug development should be addressed further.

### 2.4. Perspectives of the HIV Variants’ Open Data Use for HIV Resistance Prediction in Clinical Practice

Scientific experience leads us to the conclusions about the open data use for *HIV-1* resistance prediction.

Open data might be used for creating new computational methods because they provide the basis for training and tests sets creation. Also, due to the comparatively large number of HIV variants represented in the databases, it is possible to compare sequence data. In particular one may calculate the statistics on the highly variable regions of amino acid/nucleotide sequences for further determination of potentially resistant HIV variants [73]; it is also possible to estimate the occurrence of conserved amino acids residues close to highly variable regions; evolution studies. In addition, it the data on resistance might be processed to determine data discordance and inconsistency, and, as a result, to provide new approaches to data standardizing, and calibrating. Hence, open data might be widely applicable in academic studies.

As to the application of open data in a clinic, there are two major aspects in general use that can be considered: (1) finding the major mutations in the HIV variants’ sequences to determine the most variable/conserved nucleotides/amino acid residues in isolates from treated patients and (2) therapy outcome prediction based on the known genotype-phenotype relationships.

The HDRDB summaries, created based on the sequences collected in the database are widely used in clinics. The mutation summaries can be used to either determine major or minor mutations in population or interpret data on patients’ isolates containing major/minor mutations [74].

As to therapeutic outcome prediction, there are several points that might lead to the limitations of its use. First, there is a sort of inconsistency and noise in the data, which might lead to incorrect interpretations, if open data are used to create models, which should be used in a clinic. If we could imagine that clinical data are interpreted using models, created based on open data, the use of open data might be difficult due to the (1) existing uncertainty of the data applied for models creation and (2) unknown distances between the sequences used to train model and sequences of patients retrieved from clinic. Second, there is variability of *HIV-1* sequences retrieved from several different groups of patients, which occurs due to the prevalence of the particular HIV strain/subtype in a certain geographic region [75,76]. In our opinion, this is the most serious limitation of any data used from mixed geographic regions. However, in this case, theoretically, the superimposition of patients’ HIV variants’ sequences onto the sequences from the databases might be obtained in order to identify the cluster of sequences with relevant resistance data for model(s) creation. Third, data on the patients’ therapy regimens and their adherence to therapy are important factors influencing the outcome of therapy. Undoubtedly, these factors have to be taken into account when an outcome of therapy is predicted and they might be estimated only in the hospital where patients’ are managed. Some of these factors, in particular, adherence to therapy and concomitant diseases are very difficult to evaluate carefully for some human factors. Therefore, these limitations are related to any model directed to therapeutic outcome prediction regardless of the sequences’ data sources.

Taking into account that any *HIV-1* resistance prediction algorithm is directed to prediction of *HIV-1* resistance to a particular drug or drugs combination, two subjects should be considered in any description of *HIV-1* resistance to antiretroviral drugs: a virus (strain) and a medicine (combination of medicines). Since the *HIV-1* resistance might be partially driven by drug admission, the data on key mutations associated with resistance to a particular drug might be used to develop drugs active against strains with multiple mutations. Further, we are considering possible applications of *HIV-1* mutations’ data to new drug development algorithms.

### 2.5. Perspectives of the HIV Variants’ Open Data Use for New Drug Development

Apart from the application of the HIV proteins’ sequences for prediction of HIV genotype-phenotype relationships, the data on HIV sequences with the parameters of resistance might be used in new drug development. van Westen, G.J.P. et al. [61] have published the studies describing the use of data from HDRDB for the purposes of HIV inhibitor activity prediction. The same group of authors has reported an application of the proteochemometric approach to predict activity of a certain compound onto a mutant variant of HIV with the known sequence [77]. However, in latter study, the training set was build based on proprietary data, with further prospective validation, which exclude the use of open data in it. In particular, authors showed that the mutations in the RT sequences give the higher impact on the activity prediction comparing to chemical substructures, that were included in the model as the parameters. Therefore, one possible way to improve predictions of chemicals’ activity and/or efficacy is to use the data on genotype-phenotype relationships for different combinations of protein sequences and chemical sets.

Selection of the high conserved regions as the potentially immunogenic ones for further development of vaccines [78,79] might be one of the important applications of sequences of HIV proteins stored in databases. In that case, typically the data on HIV sequences without any data on genotype-phenotype and genotype-treatment data are used [79,80]. In particular, in the study of Yang, O.O. et al., 2015 [79] LANL database was used to select conserved regions of polypeptide sequences related to the most important for potential immunogenicity of polypeptides gag, env and nef. Further DNA vaccines were synthesized using plasmid vectors constructed based on selected sequences. Authors showed that produced vaccine has had high immunogenic characteristics for rhesus macaques with high *HIV-1* sequence coverage; the results were confirmed experimentally. In the study of Hraber, P. et al., 2015 [80] two-step computational approach, called Longitudinal Antigenic Sequences and Sites from Intrahost Evolution (LASSIE) has been proposed. The approach uses the in-house developed computational algorithm based on the metrics, reflecting “proportion of sequences sampled per time-point that mutated away” from the previous sequence state [80]. LASSIE approach has been applied to polypeptide sequences produced from *env* gene. Authors provided evidence that LASSIE may be applied for reagent selection, studies of *HIV-1* evolution for antibody/vaccine production etc. Open data on amino acid and/or nucleotide sequences is an important source for new computational approaches directed to evolutional and immunology studies potentially leading to new vaccines development. Currently, environmental HIV proteins are considered as the most important targets for these purposes.

HIV sequence data from freely available databases might be an important source for studies on *HIV-1* diversity, which, in turn, might lead to new insights in novel drug discovery. In particular, an idea that inhibitors have to be developed to target conserved regions of different viral proteins is reflected in studies dedicated to HIV proteins diversity evaluation [6,81,82,83,84]. For instance, in the study of Li, G. et al., over 12,000 *gag* sequences from LANL database were analyzed in order to determine the relationship between a number of conserved positions in *HIV-1* sequences and the probability of target to be promising for new potential antiretroviral drug discovery [80]. In particular, authors propose that the low values of intra-subtype diversity of *gag* capsid protein provide the support for considering it as a drug target. We should emphasize that currently the strategies of novel promising targets identification is in close neighborhood with the idea to combat multiple HIV targets simultaneously as well as new druggable targets search [10,83]. Probably in the near future new approaches and tools have to be developed to estimate the HIV targets, containing optimal density of conserved regions, which either can be attacked by an inhibitor or undergo potentially lethal mutations. Also, we propose that the integrating large-scale data on the HIV potentially conserved regions of HIV polypeptide sequences with the three-dimensional data on the interaction between those regions and small molecule inhibitors might be an important source for understanding key viral elements, which should be blocked and might be blocked with high probability. These data might lead to a trajectory to find new potential antiretroviral agents.

Hence, the applicability of HIV sequences’ open data is not limited to its role in HIV drug resistance prediction; it might be useful for the drug discovery approaches as well. HIV/AIDS is a great danger for humanity. Data on HIV nature and mechanisms of viral-host interaction is a key element of strategy for combatting HIV/AIDS. Information about HIV nucleotide/amino acid sequences is an important part of information about HIV nature. Therefore, the better we know all possible data on *HIV-1* structure/sequence, the higher probability is that the strategies to fighting HIV are successful. Integrated open data might be the source of information about *HIV-1* structure/sequence available worldwide, including low- and middle-income countries and providing the basis for further HIV 1 studies.

## 3. Conclusions

In our review, we have considered the basic trends of the *HIV-1* resistance/susceptibility studies: experimental methods, sources of the data on *HIV-1* sequences and its susceptibility, computational approaches. The quality of the data on *HIV-1* resistance studies is associated with the accuracy of prediction made by computational algorithms. Experimental methods used as a source for the data sets, which are widely applied for computational approaches, may affect the discordance values of the predicted results. Despite very complicated nature of HIV itself influencing the low concordance between experimental values, the data on HIV genotype-phenotype and HIV genotype-treatments relationships are the most important source for the development, validating and testing new methods of HIV drug resistance prediction. Probably, new methods integrating the data from several test systems, taking into account systematic shifts in their resistance estimates should be developed and tested. Processing the data from original scientific publications can be helpful in the task of data integration. In addition, the development of new methods for *HIV-1* resistance prediction that can take into account the differences in the results of different phenotypic assay calculated during the training process is a challenging task. Taking into account growing interest to antiretroviral vaccine development, accumulated HIV sequences’ open data have become an important source for determining the important regions for further immunology studies.

## Figures and Tables

**Table 1:** Computational algorithms for the *HIV-1* susceptibility prediction.

Name of System/Publication	Data Source *	Algorithm	No of HIV Susceptibility Levels/Another Output	Ref
Rega	Proprietary	Rule-based using Boolean expression	3 levels	[54]
HIV Grade	Proprietary	Rule-based	4 levels	[66]
Geno2Pheno	Proprietary	Decision trees; Support vector machines	Quantitative (Prediction of the FR values)	[55,56]
Retrogram	Proprietary	Rule-based	4 levels	[67]
Antiretroscan	Proprietary	Rule-based	5 levels	[68]
HIVTrePS	Proprietary	Random Forests	Estimated probability of the treatment success	[57]
EuResist	Proprietary	Combined (Bayes network Support Vector Machines, Fuzzy Logic, Case-Based Reasoning and Random Forests)	Estimated probability of the treatment success	[58]
The application of artificial neural networks for phenotypic drug resistance prediction: evaluation and comparison with other interpretation systems	Freely available (Stanford HIV resistance database)	Artificial neural networks	2 levels	[69]
Genotypic predictors of human immunodeficiency virus type 1 drug resistance	Freely available (Stanford HIV resistance database)	Decision trees, neural networks, least-squares regression (LSR), SVR, least angle regression (LARS)	3 levels	[60]
Significantly improved HIV inhibitor efficacy prediction employing proteochemometric models generated from Antivirogram data	Proprietary (training), Free available (validation)	Support vector machines	2 levels of resistance and quantitative prediction of FR value	[61]
PASS-based approach to predict *HIV-1* reverse transcriptase resistance Computational prediction of human immunodeficiency resistance to reverse transcriptase inhibitors.	Freely available (Stanford HIV resistance database)	PASS-based (modified Bayes) approach/ Set of machine learning methods	Estimated probability of the resistance occurrence/ belonging to a class of resistant variants	[62,63]

* Data source column reflects the two types of data: proprietary (not freely available) and open.

## Supplementary Materials

The following are available online: reference data sets of amino acid sequences from HIV drug resistance database containing the identifiers of NCBI Nucleotide or NCBI Protein repositories.
