# Supplementary material for: HIV Resistance Prediction to Reverse Transcriptase Inhibitors: Focus on Open Data
Source: Molecules. 2018 Apr 19;23(4):956. doi: 10.3390/molecules23040956 (PMC6017644; doi:10.3390/molecules23040956)
Supplement: Supplementary file 1 [file molecules-23-00956-s001.zip › Supplementary-materials/Figure_S1.docx]

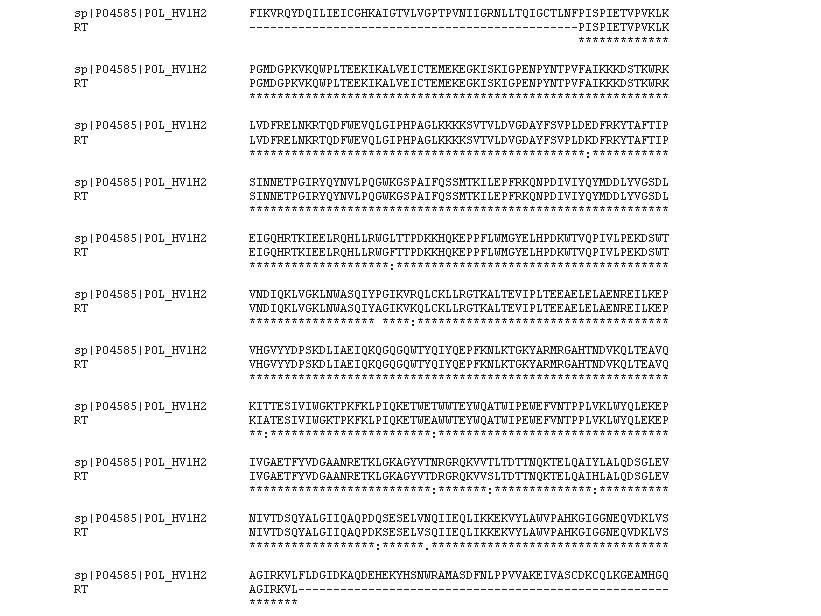


**Figure S1.** The example of alignment of the wild-type amino acid RT sequences available (1) at the Stanford HIV drug resistance database and (2) at NCBI Protein database (Accession Number: NP_057849.4).
